# Clinicopathological Observations in Acute Stroke Patients Treated with Intravenous Thrombolysis

**DOI:** 10.3390/jcm13196012

**Published:** 2024-10-09

**Authors:** Lilla Hudák, Kitti Bernadett Kovács, Zsuzsa Bagoly, István Szegedi, Viktor Bencs, Linda Lóczi, Rita Orbán-Kálmándi, Henrietta Péter-Pakó, Zsófia Fülesdi, Blanka Busi, Attila Nagy, Beáta Perjési-Kiss, László Oláh, László Csiba

**Affiliations:** 1 Department of Neurology, Faculty of Medicine, University of Debrecen, 4032 Debrecen, Hungary; 2 MTA-DE Lendület “Momentum” Hemostasis and Stroke Research Group, 4032 Debrecen, Hungary; 3 Division of Clinical Laboratory Science, Department of Laboratory Medicine, Faculty of Medicine, University of Debrecen, 4032 Debrecen, Hungary; 4 Hungarian Research Network (HUN-REN-DE) Cerebrovascular Research Group, 4032 Debrecen, Hungary; 5 Department of Radiology, Faculty of Medicine, University of Debrecen, 4032 Debrecen, Hungary; 6 Department of Pathology, Faculty of Medicine, University of Debrecen, 4032 Debrecen, Hungary; 7 Department of Health Informatics, Faculty of Health Sciences, University of Debrecen, 4032 Debrecen, Hungary

**Keywords:** ischemic stroke, autopsy, hemorrhagic transformation

## Abstract

**Background**: Ischemic stroke is a leading cause of mortality worldwide, and intravenous thrombolysis, while improving functional outcomes, still leaves a significant mortality rate. This study aimed to investigate the clinical and pathological data of thrombolysed stroke patients who subsequently died and underwent autopsy, focusing on hemorrhagic transformation (HT). **Methods**: Over a 10-year period, 1426 acute ischemic stroke patients received thrombolysis at our center, with an in-hospital mortality rate of 11.7%. Autopsies were performed on 98 of the 167 deceased patients. **Results**: HT was found in 47% of these cases, only less than half occurring within a day of thrombolysis. Significant independent predictors of HT included higher lactate dehydrogenase (LD) levels and higher INR values at admission. HT directly caused death in 30% of cases, often through herniation, while other complications (pulmonary embolism, pneumonia) were also common. **Conclusions**: These findings highlight the importance of postmortem investigations to accurately determine the incidence of HT and contributing factors. Our data indicate that in the vast majority of HT cases, the role of contributing factors other than rt-PA may be important. Of the routinely assessed clinical and laboratory parameters at admission, only LD and INR were found to be independent predictors of HT in the autopsied studied cohort.

## 1. Introduction

Acute ischemic stroke (AIS) is amongst the leading causes of death worldwide, with a permanently high mortality rate despite high-quality treatment in stroke wards [1]. According to 2023 data, it could reach 10% in the first 30 days and up to 40% by the end of the year [2]. Intravenous thrombolysis significantly increases the rate of good functional outcomes in acute ischemic stroke, but still, a considerable proportion of patients undergoing thrombolysis do not survive [3]. The rate of body and brain autopsies is decreasing worldwide, even though only autopsies can clarify the true frequencies of complications leading to death, undiagnosed tumors in the clinical phase, thromboembolic events, and thus other factors [4]. According to a retrospective analysis of 1112 patients, cardiovascular diseases notably influence disparities between ante- and postmortem diagnoses (61.7%), underlying the importance of autopsy [5]. As no observation carried out on a high number of previously thrombolysed, deceased patients undergoing body and brain autopsy is available in the literature, we aimed to investigate the clinical and pathological data of our lost patients undergoing intravenous thrombolysis and autopsy. Hemorrhagic transformation (HT) is the most feared complication of thrombolysis treatment. HT can develop after reperfusion therapy or spontaneously. HT occurs at different rates, varying between 3 and 40% depending on the definition used in different studies [6]. We aimed to find answers to the following:

1. The frequency of hemorrhagic transformation observed in the autopsies of thrombolysed AIS patients.
2. The link between HT and thrombolysis treatment (i.e., whether HT develops within a day after the treatment or later).
3. The premortem clinical differences between HT-positive and HT-negative patients.
4. Whether the direct causes of death can be attributed to HT (such as herniation) or to a complication (such as pneumonia).
5. Potential predictive markers of HT (clinical or laboratory parameters) at the admission of patients.

## 2. Materials and Methods

### 2.1. Patients

**Figure 1:**
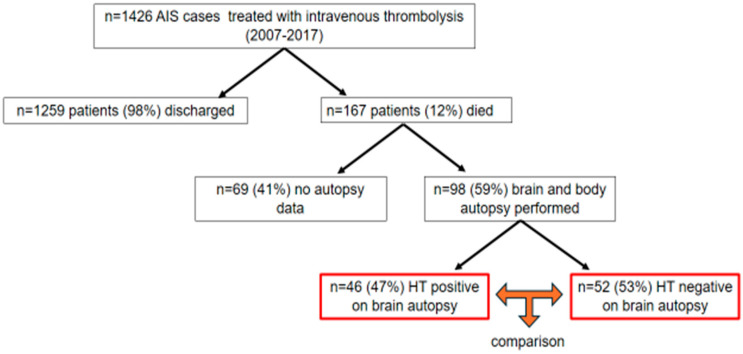
Patient recruitment flow chart. AIS, acute ischemic stroke; HT, hemorrhagic transformation.

We involved patients undergoing intravenous thrombolysis at the Department of Neurology, Faculty of Medicine, University of Debrecen, between 2007 and 2017 (neither thrombectomy nor intraarterial thrombolysis was carried out in patients). A total of 1426 treatments with intravenous thrombolysis were performed in this time period. Thrombolysis was carried out according to the protocol of the American Stroke Association (ASA), the European Stroke Initiative [7], and the European Stroke Organisation (ESO) [8]. Briefly, patients arriving within the 3 to 4.5 h time window complying with the ASA and European criteria were directly transferred to the CT laboratory. Following the CT and neurological examination, blood sampling was performed and the 1 h rt-PA infusion was administered according to the protocol. No antithrombotic treatment was initiated within 24 h after the thrombolysis, when a control cranial CT was performed to exclude HT. The presence or absence of intracranial bleeding was confirmed by a neuroradiologist (HT-positive and HT-negative group). Patients after the thrombolysis were transferred to the stroke ward where multiparametric monitoring (blood pressure, blood oxygen saturation, transcranial ultrasound, arterial blood gas, and so on) was carried out. Patients received antipyretics, antibiotics and antithrombotics (deep venous thrombosis prophylaxis), if needed. The average time of hospitalization was 16.71 ± 16.36 days at our department before death. The in-hospital mortality of the 1426 patients who underwent intravenous thrombolysis was 11.7%. According to Hungarian health law, an autopsy must be performed on every deceased person in a hospital. An autopsy may be waived if the clinician finds the cause of death to be clear and the relatives request the waiver of the autopsy, or if the deceased had objected to the autopsy during their lifetime and the death was of natural origin. In the case of our patients, we waived the autopsy when the above conditions were met. We did not use the data of some autopsied patients because the data were incomplete. We had the chance to compare the body and brain autopsy results with the clinical data in 98 cases (59%) out of the 167 deceased patients (Figure 1). The body autopsy was carried out the following day after death, and brain autopsy took place after one-week formalin fixation of the brain. The latter was performed by a neuropathologist. The neuropathologist determined whether hemorrhagic transformation was present or not during the slicing of the brain through visual inspection. Hemorrhagic transformations were grouped according to the ECASS radiological classification: HI 1 as small petechiae along the margins of the infarct, and HI 2 as more confluent petechiae within the infarcted area, but without space-occupying effect. PH 1 was defined as a clot not exceeding 30% of the infarcted area with some mild space-occupying effect. PH 2 represented dense blood clot(s) exceeding 30% of the infarct volume with significant space-occupying effect [9].

### 2.2. Statistical Analysis

Statistical analysis was performed using Stata v13 software, the Statistical Package for Social Sciences (SPSS, Version 26.0, Chicago, IL, USA), and GraphPad Prism 9.0 (GraphPad Prism Inc., La Jolla, CA, USA). The normality of the data was studied using the Shapiro–Wilk test. Student’s *t*-test or the Mann–Whitney U test was performed for independent two-group analyses. Differences between categorical variables were assessed by χ^2^ test or by Fisher’s exact where appropriate. Receiver operating characteristic (ROC) curves were built by plotting sensitivity vs. 1-specificity and calculating the area under the curve (AUC). Youden’s J statistics were used to calculate optimal threshold values. Sensitivity, specificity, positive predictive values (PPVs), and negative predictive values (NPVs) were calculated using contingency tables and χ^2^ test or Fisher’s exact at statistically optimal threshold values. Binary backward logistic regression models were used to determine independent predictors of HT in the studied cohort. Adjustments of the models were based on the results of univariate statistical analyses of baseline characteristics between groups. Results of the logistic regression analysis were expressed as odds ratio (OR) and 95% confidence interval (CI). A *p*-value of <0.05 was considered statistically significant.

## 3. Results

### 3.1. Autopsy Results and Baseline Characteristics

HT was identified in nearly half of the cases at autopsy (46, 47% HT-positive vs. 52, 53% HT-negative cases). A total of 19 HT complications, 19/46, were already seen on the 24 h control CT and one was described on a follow-up CT. Interestingly, less than half of HT cases occurred within a day after thrombolysis (41% 19/46), while the majority of HT events (59% 27/46) developed later, between day 1 and the event of death. Out of the 98 patients, a second follow-up CT was performed in 25 cases due to clinical deterioration. In one patient, HT was found that was not visible on the post-lysis control CT. In the remaining 24 cases, the follow-up CT confirmed the findings of the post-lysis control CT (presence or absence of HT). We categorized the distribution of HT according to the ECASS criteria (Table 1). 

The direct causes of death in these cases were frequently attributed to the HT event itself or its consequences (Table 2). Based on the results of body and brain autopsy, a direct CNS complication or herniation was responsible for death in 29 cases (30% of the total cohort), while pneumonia, pulmonary embolism, and cardiorespiratory failure were the causes of death in the remaining cases.

Two cases of malignant cancers were discovered (pancreatic adenocarcinoma, neuroendocrine tumor) during the autopsies that had not been diagnosed previously due to subtle symptoms only.

The investigated baseline clinical and laboratory parameters in the HT-positive and HT-negative cases are presented in Table 3. Interestingly, few laboratory parameters showed a difference between the two groups at admission. HT-positive patients had significantly higher lactate dehydrogenase (LD) levels at admission as compared to HT-negative patients (median: 272, IQR: 217–444 vs. 204, IQR: 176–264 U/L, *p*: 0.0011). In addition, significantly lower platelet count was observed in HT-positive vs. HT-negative patients before thrombolysis (median: 184, IQR: 150–227 vs. 223, IQR: 171–264 G/L, *p*: 0.0106), together with a significantly longer prothrombin time (PT) and higher INR values (median: 1.1, IQR: 0.9–1.1 vs. 0.9, IQR: 0.9–1.0, *p*: 0.0429).

We investigated whether the antithrombotic treatment used before and after the onset of ischemic stroke influenced the risk of hemorrhagic transformation of the developing ischemic lesion. Concerning the use of different antithrombotic therapy combinations before thrombolysis (antiplatelets and/or anticoagulants), no significant difference was observed between the HT-positive and HT-negative group (χ^2^ test: *p*:0.089), but it is to be noted that a hemorrhagic tendency was present among those anticoagulated. After the event of stroke, no significant difference was observed between the group receiving therapeutic or prophylactic anticoagulant therapy in terms of HT rates (*p* = 0.808). However, it is important to note that of the 98 cases included, HT had been already clinically detected in 20 cases on day 1; therefore, more patients received prophylactic anticoagulants for DVT prophylaxis in the HT-negative group. Looking separately at the 26 cases (treated as pure ischemic lesions during clinical course) with clinically not recognized HT at brain autopsy compared to the 52 cases without bleeding at brain autopsy, there was no significant association between antithrombotic therapy and bleeding (χ^2^ test *p* = 0.945).

### 3.2. Independent Predictors of HT in the Studied Autopsied Cohort

**Figure 2:**
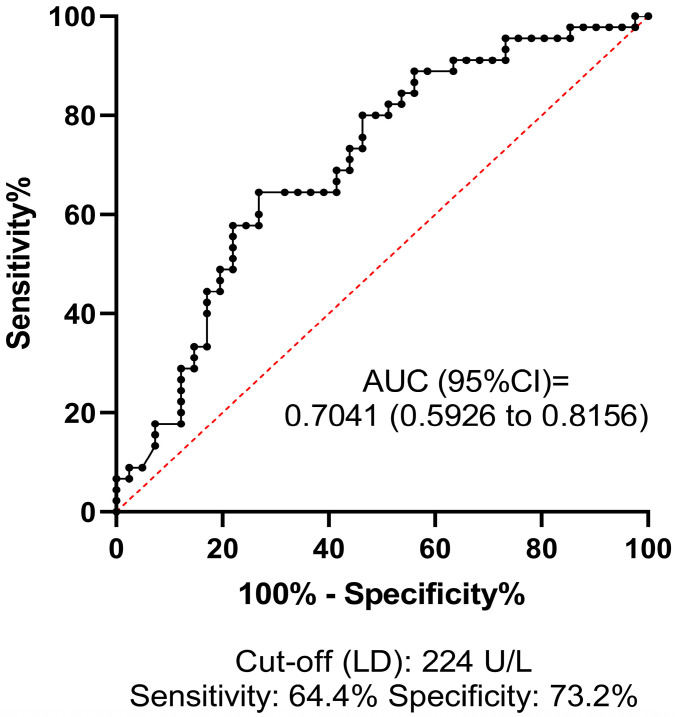
Receiver operator characteristics (ROCs) curve of lactate dehydrogenase (LD) at admission for estimating hemorrhagic transformation in AIS patients treated with intravenous thrombolysis. ROCs curve and descriptive statistics including best cut-off value as determined by the Youden’s J statistics are depicted for LD. AUC, area under the curve; CI, confidence interval.

**Figure 3:**
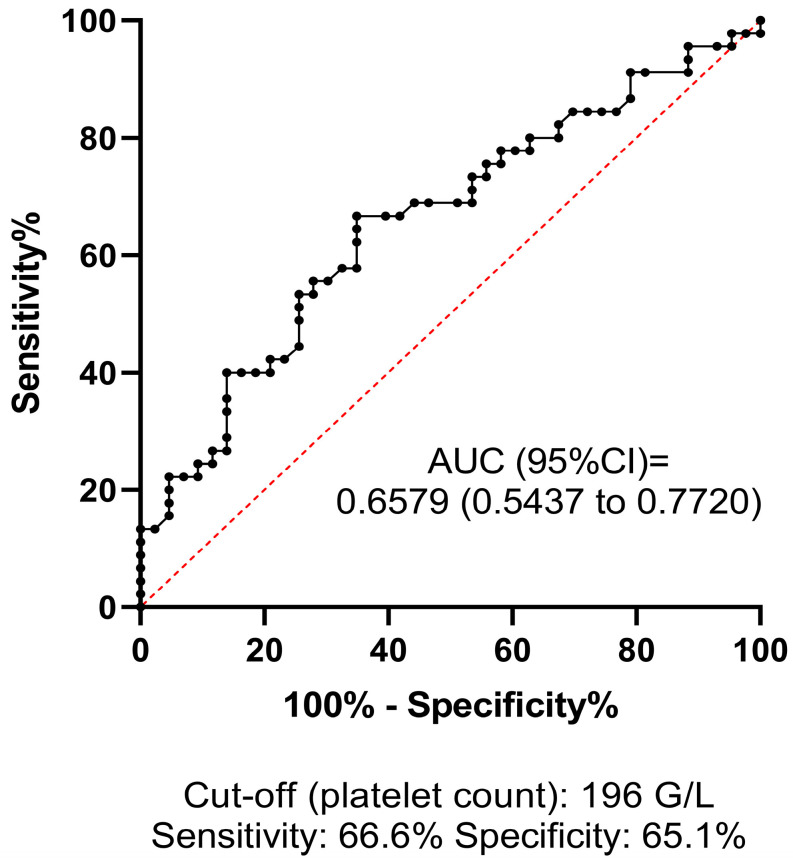
Receiver operator characteristics (ROCs) curve of platelet count at admission for estimating hemorrhagic transformation in AIS patients treated with intravenous thrombolysis. ROCs curve and descriptive statistics including best cut-off value as determined by the Youden’s J statistics are depicted for platelet count. AUC, area under the curve; CI, confidence interval.

**Figure 4:**
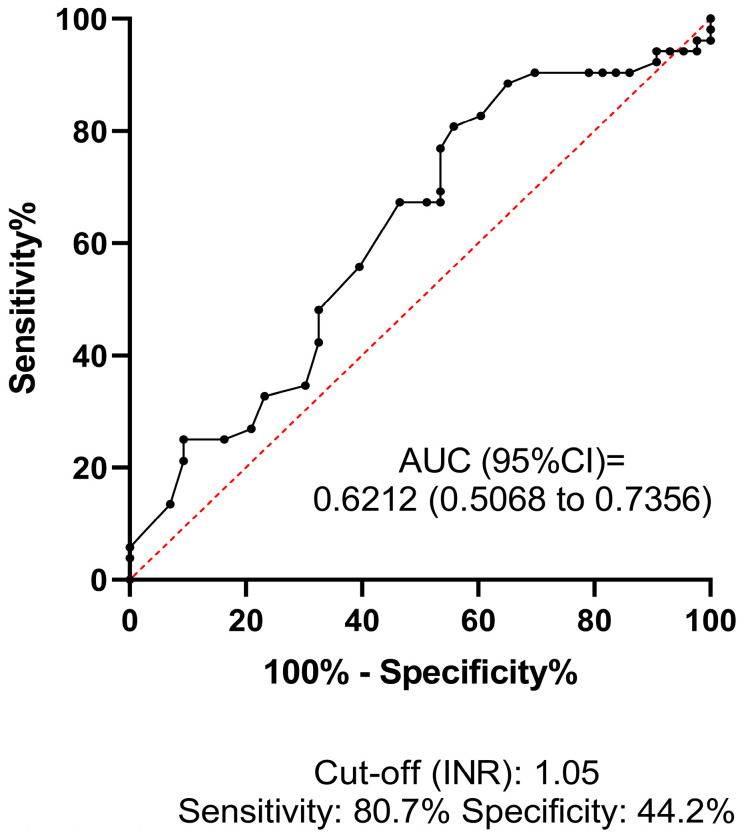
Receiver operator characteristics (ROCs) curve of INR at admission for estimating hemorrhagic transformation in AIS patients treated with intravenous thrombolysis. ROCs curve and descriptive statistics including best cut-off value as determined by the Youden’s J statistics are depicted for INR. AUC, area under the curve; CI, confidence interval; INR, international normalized ratio.

ROC analysis was performed to investigate the predictive value and diagnostic efficacy of all baseline test parameters that differed significantly between groups according to univariate statistical analyses. The best AUC of ROC for predicting HT was 0.7041 (95% CI: 0.5926–0.8156) for LD, at the optimal statistical threshold of 224 U/L (sensitivity: 64.4%, specificity: 73%) (Figure 2). The AUC of ROC for platelet count was 0.6579 (95% CI: 0.5437–0.7720), at the optimal statistical threshold of 196 G/L (sensitivity: 66.6%, specificity: 65.1%) (Figure 3). This result suggests that a relatively low platelet count (<196 G/L), despite being within the reference range (150–400 G/L), may increase the risk of HT in AIS patients receiving thrombolysis. Based on the optimal threshold value as defined by the Youden index (INR: 1.05), the best sensitivity was provided by the INR parameter (80.7%, Figure 4), although the low AUC value suggests poor overall test performance.

Using a binary backward logistic regression model (including age, sex, NIHSS on admission, hypertension, INR ≥ 1.05, LD ≥ 224 U/L, platelet count ≥ 196 G/L, ASAT, ALT, GGT, creatine kinase, hsCRP, creatinine), only LD and INR remained as significant, independent predictors of HT in the studied autopsied cohort (OR: 4.68, 95% CI: 1.57–14.00, *p* = 0.006 and OR: 6.23, 95% CI: 1.55–25.13, *p* = 0.010, respectively; Table 4).

## 4. Discussion

HT is a (symptomatic or asymptomatic) complication in case of acute ischemic stroke that may occur spontaneously but can be a considerable consequence of intravenous thrombolysis. According to Jensen et al. intravenous thrombolysis approximately doubled the frequency of HT (HR: 2.08 [95% CI, 1.28–3.40]) [10]. Pande et al. followed the frequency of hemorrhagic complications in thrombolysed patients by imaging techniques and found it to be 36.6% [11]. In the ECASS II study (thrombolysed patients), the rate of hemorrhagic transformation was 35.6% [12]. The actual frequency of HT in the above mentioned studies was probably even higher, as justification of HT was based on the results of the 24 h control and a further cranial CT performed only in case of clinical deterioration. Therefore, in asymptomatic HT patients—unless a second cranial CT scan was performed for any reason—the hemorrhagic complication remained unnoticed. However, brain autopsy of the patients suffering from ischemic stroke provides reliable information on the actual frequency of hemorrhagic transformation. Our working group had previously investigated the brain autopsy results of ischemic stroke patients who did not undergo intravenous thrombolysis, and the rate of hemorrhagic transformation was 29–38% [4,13,14]. Rt-PA has been shown to disrupt blood and brain barrier (BBB) integrity via several ways, including LDL receptor-related protein (LRP) expression on the endothelial cells, microglia, and astrocytes [15], increasing plasma kallikrein [16] and platelet-derived growth factor-CC (PDGF-CC) activation [17]. Shi et al. demonstrated that rt-PA also mobilizes immune cells exacerbating HT after ischemic stroke [18]. In our current study, the frequency of HT was only 19% on the control cranial CT performed 24 h after the intravenous thrombolysis (19/98), while the autopsy found 47% (46/98). The 19% of HT detected on the first day does not significantly differ from the observation of the ECASS-I study: they found some kind of HT in 48 cases out of 264 acute ischemic (not thrombolysed) stroke patients (18%) [9].

According to a recent multicenter retrospective study (32,375 patients), the rate of symptomatic and asymptomatic intracranial hemorrhage after intravenous thrombolysis is 17.5% (95% CI, 17.0–18.0) [19]. The meta-analysis of Honig et al. systematically reviewed the incidence, predictors, and outcomes of hemorrhagic transformation (HT) in 65 studies with 17,259 patients with acute ischemic stroke using ECASS-II criteria [20]. The overall prevalence of HT was 27%, with 32% occurring in patients receiving IV-tPA compared to 20% in those who did not receive it. Our observation is that the 20% HT rate 24 h after thrombolysis increased to 47% upon brain autopsy, which cannot be attributed either to the immediate action of rt-PA (half-life is 4–6 min) or to the prolonged activation of the plasminogen–plasmin system, which can last several hours [21,22]. Therefore, other factors also play an important role in the development of HT between the 24 h control cranial CT and death. One of our most important findings is as follow: a significant portion of HT (59% 27/46) is not directly related to the lysis (they are not visible on the control CT after lysis (first day) but are detected during autopsy). The AHA 2021 guideline, in the section on secondary prevention, mentions (with 2b) that “patients with ischemic stroke and the treatment that includes anticoagulant therapy, CT or MRI of the brain before therapy is started may be considered to assess for hemorrhagic transformation and final size of infarction”. But we believe that instead of “may be considered”, it would be more appropriate to “recommend” a CT/MRI scan before starting or restarting antithrombotic therapy. According to our results, a low platelet count and prolonged PT are factors provoking HT and an unfavorable clinical outcome. Our statements are supported by other researchers as well; for example, Cheng et al. reported that low platelet levels were independently associated with HT in non-AF patients [23]. Domingo et al. found that patients with low platelet counts had increased mortality compared with patients with normal platelet counts following desobliteration for large vessel occlusion [24]. Mustanoja at el. investigated 636 young patients (median age was 42.9 year) and found no significant correlation with gender, age, prehospital oral anticoagulant, antiplatelet or statin therapy and blood glucose level upon admission but the probability of HT increased with—among others—low platelet count [25]. Prodan et al. reported that lower levels of coated-platelets are associated with the presence of early HT in patients with non-lacunar ischemic stroke [26]. On the other hand, using a binary backward regression model, only LD and INR remained in the statistical model as independent predictors of HT, which reflects that although the balance of hemostasis is most likely an important contributor to HT occurrence, other potential contributing factors might play key roles. According to a 2023 study that examined LDH levels in patients with subarachnoid hemorrhage, admission LDH levels were significantly higher in patients with unfavorable neurological outcomes. The highest LDH value during the ICU stay (OR 1.004 [95% CI 1.002–1.006]) was independently associated with poor outcomes [27]. A Chinese group investigated the relationship between LDH levels and outcomes in ischemic stroke. Their results indicated that patients in the highest LDH quartile had a higher risk of all-cause death [(HR), 2.23; 95% confidence interval (CI), 1.27–3.90], and a higher proportion of mRS scores 3–6 [odds ratio (OR), 1.54; 95% CI, 1.26–1.90] and mRS scores 2–6 (OR, 1.56; 95% CI, 1.32–1.84) at 3 months. High lactate dehydrogenase was associated with adverse outcomes in patients with acute ischemic stroke or transient ischemic attack [28]. As—according to our observation—the vast majority of the HT develops between the 24 h control cranial CT and death, we suggest performing a further cranial CT before discharging the patient independent of their neurological status to exclude HT, as its presence may influence the initiation of an antithrombotic or antiplatelet therapy. The current European guideline does not contain strong recommendations concerning the regular checking of platelet count in case of acute ischemic stroke or after thrombolysis. However, our findings suggest the importance of a decreased platelet count to be even higher than previously thought. Platelet count is to be checked more often after thrombolysis and medications decreasing it should be avoided. Multicenter prospective studies can verify our suggestions. Two malignant tumors were detected upon body autopsy that remained unnoticed during the clinical phase due to lack of symptoms. Systemic tumors can provoke cerebral ischemia by affecting/influencing the coagulation cascade. According to previous observations, 3–5% of patients were diagnosed with a malignancy after suffering an ischemic stroke [29]. The most common tumors in patients who had a stroke are lung, gastrointestinal, and breast tumors [30]. Thrombolytic therapy can provoke not only space-occupying HT but also malignant brain edema and herniation, leading to death [31]. According to our observations HT was the direct cause of death only in 30% of the patients (via herniation), the rest of our patients were lost due to other complications, mainly pneumonia. The stroke victims were bed-ridden with hypoventilation, possible aspiration with pneumonia, and other respiratory infections. VBI strokes can impair the respiratory centers in the brainstem responsible for controlling breathing. Dysphagia is also a common post-stroke, increasing the risk of aspiration, which can lead to aspiration pneumonia—a major cause of morbidity and mortality in stroke patients. [32]. The considerable role of pneumonia as a cause of death is supported by the observations of Katzan et al., who investigated more than 11,000 acute ischemic stroke patients and found that pneumonia conferred a threefold increased risk of 30-day death [33]. According to an American study, 47% of critically ill patients who had a stroke developed pneumonia [34]. Strokes can lead to cardiac complications (arrhythmias, myocardial infarction, and heart failure). A stroke can trigger a cascade of autonomic dysfunctions leading to cardiovascular instability. Strokes often result in increased sympathetic nervous system activity, causing hypertension, arrhythmias, and other cardiovascular complications. Stress-induced cardiomyopathy, also known as “broken heart syndrome”, can also occur following a stroke [35]. In our study, cardiorespiratory insufficiency was significantly higher in a non-HT group than in the HT group. Our results should be interpreted in the context of its strengths and limitations. A major limitation of this study is that it was restricted to a single center, which limited the sample size; however, this assured unified patient care and autopsy practices. A second limitation is that 41% of the deceased patients did not undergo autopsy in our center on the basis of the request of the relatives. The data were collected over a 10-year period (2007–2017) in which this study focused exclusively on patients who received intravenous thrombolysis, excluding those who underwent thrombectomy or intra-arterial thrombolysis. Changes in stroke management protocols, thrombolysis techniques, and supportive care over this period might have influenced the outcomes, making it difficult to attribute findings to a consistent treatment approach. Lastly, this study did not perform a detailed subgroup analysis based on the severity or subtypes of hemorrhagic transformation. Grouping all types of HT together might have masked specific factors relevant to different HT subtypes.

## 5. Conclusions

Our results highlight the importance of postmortem investigations.

- The results of autopsy findings of our patients confirm the high rate of HT after intravenous thrombolysis.
- The majority of HTs were not directly related timely to the t-PA lysis.
- This observation suggests that when considering potential anticoagulant therapy after lysis, a new CT scan should be performed even if the initial post-lysis CT showed no HT and the patient’s clinical condition has not worsened.
- According to the autopsy data, in the majority of lysis patients who suffered HT complications, HT was not the direct cause of death (only in 39%), but rather other complications (such as myocardial infarction, pneumonia). This observation also highlights the importance of more effective management of associated diseases.
- Further large-scale prospective studies are needed to confirm the observation that elevated admission LD levels increase the risk of HT.
- We would like to express our thanks to Tibor Hortobágyi for performing the brain dissections.

## Figures and Tables

**Table 1:** Distribution of HT characteristics according to ECASS criteria. Based on control CT one day after thrombolysis.

Variables (*N*:19)	HI1	HI2	PH1	PH2
Number of individuals, *n* (%)	3 (16)	4 (21)	4 (21)	8 (42)

**Table 2:** Causes of death in the autopsied study cohort.

Variables	HT-Negative	HT-Positive	*p*
Number of individuals, *n*	52	46 *	<0.001
Causes of death, *n* (%)			
Herniation	11 (21)	18 (39)	0.052
Pulmonary embolism	0 (0)	2 (4)	1.000
Myocardial infarction and/or severe heart failure	14 (27)	8 (17)	0.259
Pneumonia	18 (35)	17 (37)	0.809
Sepsis	2 (4)	0 (0)	0.497
Cardiorespiratory insufficiency	7 (13)	0 (0)	0.013

HT, hemorrhagic transformation. * Specific cause of death could not be determined in case of one patient.

**Table 3:** Baseline characteristics of the study population.

Variables	HT-Positive	HT-Negative	*p*
Number of individuals, *n*	46	52	<0.001
Age, year	75.8 ± 8.9	72.5 ± 10.4	0.100
Male sex, *n* (%)	24 (52)	30 (58)	0.685
Active smoker, *n* (%)	9 (20)	15 (29)	0.286
Laboratory parameters			
Serum glucose, mmol/L	7.6 (6.6–9.1)	8.1 (6.2–9.7)	0.987
Urea, mmol/L	6.3 (5.3–7.8)	6.6 (4.7–8.4)	0.786
Creatinine, µmol/mL	80 (63–96)	81 (63–107)	0.644
eGFR, mL/min	69 (54–91)	71 (54–89)	0.767
Creatine kinase, U/L	76 (44–128)	69 (54–89)	0.674
ASAT, U/L	21 (16–27)	18 (15–23)	0.117
ALT, U/L	16 (12–20)	15 (11–24)	0.837
GGT, U/L	36 (18–65)	32 (22–47)	0.531
LD, U/L	272 (217–444)	204 (176–264)	0.0011
Triglyceride, mmol/L	1.1 (0.7–1.5)	1.1 (0.86–1.6)	0.376
Total cholesterol, mmol/L	4.6 (4.1–5.7)	4.9 (3.9–5.8)	0.355
hsCRP, mg/L	2.0 (0.9–3.5)	3.5 (1.5–5.8)	0.810
WBC, G/L	8.1 (6.5–10.3)	8.0 (7.0–9.9)	0.915
Hemoglobin, g/L	138 (126–150)	136 (121–147)	0.514
Platelet count, G/L	184 (150–227)	223 (171–264)	0.011
PT, s	9.1 (8.2–9.5)	8.4 (8.0–9.0)	0.008
INR	1.1 (0.9–1.1)	0.9 (0.9–1.0)	0.043
APTT, s	29.5 ± 3.9	28.4 ± 3.5	0.141
TT, s	18.9 (17.7–20.2)	19.2 (18.0–20.4)	0.380
NIHSS before thrombolysis	15.0 (10.8–18.3)	13.5 (8.5–18.8)	0.682
NIHSS after thrombolysis	16.0 (10.8–19.0)	14.0 (8.0–19.0)	0.478
Days of hospitalization	16 ± 15	17 ± 17	0.636

Continuous variables are expressed as mean ± standard deviation or median (interquartile range). Categorical variables are indicated as number (percentage). ALT, alanine transaminase; APTT, activated partial thromboplastin time; ASAT, aspartate transaminase; eGFR, estimated glomerular filtration rate; GGT, gamma-glutamyl transferase; hsCRP, high-sensitivity C-reactive protein measurement; HT, hemorrhagic transformation; INR, international normalized ratio; LD, lactate dehydrogenase; *n*, number; NIHSS, National Institutes of Health Stroke Scale; PT, prothrombin time; TT, thrombin time; WBC, white blood cell.

**Table 4:** Independent predictors of hemorrhagic transformation in the studied cohort.

	OR	95% CI	*p*
LD (≥224 U/L)	4.68	1.57–14.00	0.006
INR (≥1.05)	6.23	1.55–25.13	0.010

Last step of backward multiple regression analysis is provided. Backward multiple regression model included age, sex, NIHSS on admission, INR ≥ 1.05, LD ≥ 224 U/L, platelet count ≥ 196 G/L, ASAT, ALT, GGT, creatine kinase, hsCRP, and creatinine. 95% CI, 95% confidence interval; INR, international normalized ratio; LD, lactate dehydrogenase; NIHSS, National Institutes of Health Stroke Scale; OR, odds ratio.

## Data Availability

Data are available upon reasonable request. All data relevant to the study are included in the article. The data that support the findings of this study are available from the corresponding author, L.C. (University of Debrecen Faculty of Medicine Department of Neurology, csiba@med.unideb.hu), upon reasonable request.
